# Mendelian randomization reveals the impact of diet on infertility in men and women

**DOI:** 10.3389/fendo.2024.1376800

**Published:** 2024-04-23

**Authors:** Xiangyu Chen, Congzhe Ren, Changgui Wu, Xiaoqiang Liu

**Affiliations:** Department of Urology, Tianjin Medical University General Hospital, Tianjin, China

**Keywords:** DIETS, male infertility, female infertility, mendelian randomization, GWAS

## Abstract

**Background:**

Although studies on the effects of diet on fertility has progressed, some cumulative evidence has piled against popular hypotheses. The aim of our study was to investigate the effects of 31 diets including 23 individual dietary intakes and 8 dietary habits on infertility in men and women.

**Methods:**

The datas of diets and infertility were collected from genome-wide association studies (GWAS). Mendelian randomization (MR) methods were used to analyze causal relationships. Multivariate MR (MVMR) adjusted for the effects of other exposures on causality. And MR-Egger, Cochran’s Q, radial MR, and MR-PRESSO tests were employed to assess heterogeneity and horizontal pleiotropy.

**Results:**

Our study found that coffee intake (OR, 3.6967; 95% CI, 1.0348 – 13.2065; P = 0.0442) and cooked vegetable intakes (OR, 54.7865; 95% CI, 2.9011 – 1030.5500; P = 0.0076) increased the risk of male infertility. For women, beer was a risk factor for infertility (OR, 4.0932; 95% CI, 1.8728 – 8.9461; P = 0.0004); but processed meat was negatively associated with infertility (OR, 0.5148; 95% CI, 0.2730 – 0.9705; P = 0.0401). MVMR demonstrated selenium as a protective factor against female infertility (OR, 7.4474e-12; 95% CI, 5.4780e-22 – 1.0125e-01; P = 0.0314).

**Conclusion:**

We found the causal relationships between four diets and infertility. We look forward to more high-quality epidemiologic studies to prove our conclusions.

## Introduction

Infertility is defined as the failure to achieve a clinically recognized pregnancy after 12 months or more of regular unprotected sexual intercourse (1). Globally, infertility affects 15% of couples at reproductive age (2). According to reports, 33–41% of cases of infertility are caused exclusively by a female factor, 25–39% by a male factor, and 9–39% by a combination of both male and female factors (3). Infertility can cause severe psychological distress to the patient, as well as great distress and economic burden to society (4).

Current treatment options for infertility include assisted reproductive technologies (ART) including ovarian stimulation with or without intrauterine insemination (IUI) and *in vitro* fertilization (IVF). Despite increased ART use, the results for amelioration of infertility are still poor, with an average of only one in five cycles resulting in a live birth (5). Given the high prevalence of impaired fertility, coupled with the high financial cost of infertility treatment, the impact of lifestyle factors on infertility is increasingly being considered. Being overweight or obese plays a significant role in the fertility status of a couple (6). Although diet and obesity are undoubtedly related, there is growing evidence that diet may independently influence fertility. This effect depends on the quantitative and qualitative impact of diet, including calories per macronutrient, and specific proteins, carbohydrates, and certain acid-spectrum fatty acids (7, 8). For men, for example, high-fat diets not only have a strong negative impact on sperm parameters, but are also associated with smaller testes, lower seminal vesicle and epididymal quality. Most importantly, high-fat diets are associated with lower fertilization success rates (9, 10). In addition, following a “fertility diet” may favorably impact the fertility of otherwise healthy women, as well as preventing most cases of infertility caused by ovulatory disorders (11). Although the impact of diet on reproductive performance has been increasingly recognized in recent years, there are no dietary guidelines for fertility (12). The aim of our study was to explore the causal link between 31 different dietary plans and infertility using Mendelian randomization (MR) methods. In addition, we explored the effects of diet on infertility in men and women, with a view to providing guidance on dietary choices for couples of childbearing age; and to provide a basis for investigating the mechanisms of sex differences while probing the relationship between diet and infertility.

MR is an epidemiological method that uses genetic variants to simulate an exposure and predict its causal association with an outcome. The main limitation of observational studies and randomized trials is the inadequate validation of confounders, leading to biased estimates of causality. In contrast, genetic variation is a lifetime constant, independent of confounding effects of the environment (13, 14).

## Methods

### Data sources

For the causal analysis, diet was considered as the exposure variable, and infertility in men and women as the outcome variable. The genetic variants of dietary intake comprised output from the genome-wide association studies (GWAS) pipeline using Phenome-wide Scan Analysis Tools (PheSANT)-derived variables of the UK Biobank cohort of about 500,000 individuals (15). The list of 31 dietary intakes included in the analysis consisted of 23 individual dietary intakes and 8 dietary habits. 23 individual dietary intakes include bacon, beef, bread, cereal, cheese, coffee, cooked vegetable, dried fruit, fresh fruit, lamb, milk, non-oily fish, oily fish, pork, poultry, processed meat, raw vegetable, salted nuts, salted peanuts, tea, unsalted nuts, unsalted peanuts, and yogurt; and 8 dietary habits include average weekly beer intake, average weekly red wine intake, average weekly spirits intake, never eat dairy products, never eat eggs or foods containing eggs, never eat sugar or foods/drinks containing sugar, never eat wheat products, and eat eggs, dairy, wheat, and sugar. Upon participant recruitment, a touchscreen questionnaire used in UK Biobank asked twenty-nine questions about diet, most of which gathered information about the mean frequency of consumption of food and food groups over the past year.

Genetic data for the outcome phenotype including male infertility (680 cases and 72,799 controls) and female infertility (6,481 cases and 68,969 controls) were collected from the FinnGen Consortium.

### Instrumental variables selection

A reliable MR study must fulfill three basic assumptions: (1) the genetic variations and the exposure of interest are closely associated; (2) the genetic instruments have no impact on the outcome and are unaffected by any confounding factors; and (3) the exposures of interest are the only factors that mediate the effects of instrumental variables (IVs) on the outcomes. We specified strict single-nucleotide polymorphisms (SNPs) filtering criteria. SNPs that were associated with diet and infertility were extracted with a GWAS threshold (p<5×10^-8^). Due to the low number of SNPs at the original threshold after screening for the intake of bacon, milk, yogurt, salted peanuts, unsalted peanuts, salted nuts, spirits intake, unsalted nuts, never eat dairy, never eat wheat, and never eat eggs, we chose a relaxed threshold (p<5×10^-6^). Using the linkage disequilibrium (LD) clustering method, SNPs with LD were identified and removed (clumping: r^2^ = 0.001, kb = 10,000). We also searched the PhenoScanner website (http://www.phenoscanner.medschl.cam.ac.uk/information/) to identify IVs included in the study and to exclude SNPs associated with outcomes. Lastly, SNPs with weak IV bias were assessed using F statistics. The formula used to compute F statistics was F=beta^2^/se^2^. In the MR analysis, only SNPs with F statistic values greater than 10 were considered present.

### Statistical analysis

Inverse variance-weighted (IVW), weighted median (WM), and MR-Egger tests were used for two-sample Mendelian randomization (TSMR) analysis. For IVW data, we used meta-analysis to combine the Wald estimates for each SNP and obtain an overall estimate of the effect of exposure on outcome. This was considered the strongest MR analysis method (16). And the multivariate MR (MVMR) method adjusts for the effect of other exposures on outcomes. In sensitivity analysis, Cochran’s Q test was used to quantify the heterogeneity between IVs. The MR-Egger intercept test uses the intercept term to evaluate pleiotropy, ensuring genetic variants independently linked to exposure and outcome are employed for the computations. Additionally, we employed the MR pleiotropy residual sum and outlier (MR-PRESSO) and radial MR tests to identify horizontal pleiotropy and outliers. A distortion test was used to determine the results before and after correcting for outliers. Finally, a “leave-one-out” analysis was performed to assess the stability of the results, implying that one SNP was sequentially excluded to estimate whether it biased the results.

## Results

### Diet and infertility in men

**Figure 1:**
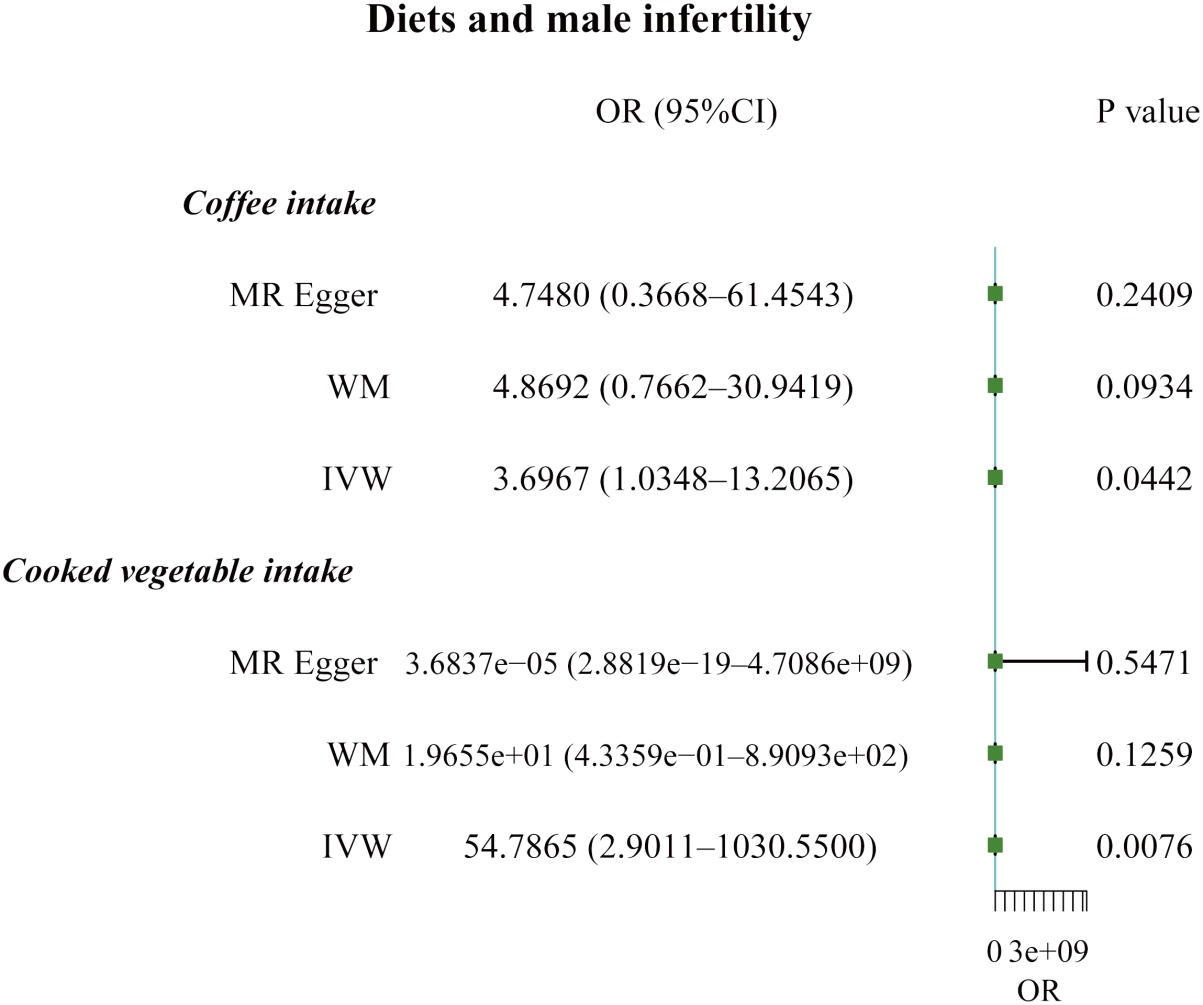
Forest plot of the causal associations between coffee and cooked vegetable intakes, and male infertility.

The F-statistic values for all IVs were higher than 10, avoiding the risk of weak instrumental bias (**Table 1**). The results of the TSMR analysis indicated that coffee intake increased the risk of male infertility (Odds Ratio [OR], 3.6967; 95% Confidence Interval [CI], 1.0348 – 13.2065; P = 0.0442). Furthermore, there was a significant positive correlation between cooked vegetable intake and male infertility (OR, 54.7865; 95% CI, 2.9011 – 1030.5500; P = 0.0076) (**Figures 1**;, **2A** **;** **Supplementary Table 1**).

**Table 1:** Detailed information about data sources of diets and infertility.

Traits	GWAS ID	SNPs	Sample size	Year	P value	F-statistics	Pubmed ID (or URL)
Bacon intake	ukb-b-4414	9,851,867	64,949	2018	5 × 10^-6^	27.9	http://app.mrbase.org
Beef intake	ukb-b-2862	9,851,867	461,053	2018	5 × 10^-8^	20.3	http://app.mrbase.org
Beer intake	ukb-b-5174	9,851,867	327,634	2018	5 × 10^-8^	25.4	http://app.mrbase.org
Bread intake	ukb-b-11348	9,851,867	452,236	2018	5 × 10^-8^	25.9	http://app.mrbase.org
Cereal intake	ukb-b-15926	9,851,867	441,640	2018	5 × 10^-8^	22.1	http://app.mrbase.org
Cheese intake	ukb-b-1489	9,851,867	451,486	2018	5 × 10^-8^	27.8	http://app.mrbase.org
Coffee intake	ukb-b-5237	9,851,867	428,860	2018	5 × 10^-8^	22.7	http://app.mrbase.org
Cooked vegetable intake	ukb-b-8089	9,851,867	448,651	2018	5 × 10^-8^	22.7	http://app.mrbase.org
Dried fruit intake	ukb-b-16576	9,851,867	421,764	2018	5 × 10^-8^	31.9	http://app.mrbase.org
Fresh fruit intake	ukb-b-3881	9,851,867	446,462	2018	5 × 10^-8^	33.4	http://app.mrbase.org
Lamb intake	ukb-b-14179	9,851,867	460,006	2018	5 × 10^-8^	26.1	http://app.mrbase.org
Milk intake	ukb-b-2966	9,851,867	64,943	2018	5 × 10^-6^	20.8	http://app.mrbase.org
Non-oily fish intake	ukb-b-17627	9,851,867	460,880	2018	5 × 10^-8^	25.6	http://app.mrbase.org
Oily fish intake	ukb-b-2209	9,851,867	460,443	2018	5 × 10^-8^	27.4	http://app.mrbase.org
Pork intake	ukb-b-5640	9,851,867	460,162	2018	5 × 10^-8^	21.3	http://app.mrbase.org
Poultry intake	ukb-b-8006	9,851,867	461,900	2018	5 × 10^-8^	23.0	http://app.mrbase.org
Processed meat intake	ukb-b-6324	9,851,867	461,981	2018	5 × 10^-8^	27.8	http://app.mrbase.org
Raw vegetable intake	ukb-b-1996	9,851,867	435,435	2018	5 × 10^-8^	29.6	http://app.mrbase.org
Red wine intake	ukb-b-5239	9,851,867	327,026	2018	5 × 10^-8^	29.8	http://app.mrbase.org
Salted nuts intake	ukb-b-15960	9,851,867	64,949	2018	5 × 10^-6^	24.1	http://app.mrbase.org
Salted peanuts intake	ukb-b-1099	9,851,867	64,949	2018	5 × 10^-6^	28.5	http://app.mrbase.org
Spirits intake	ukb-b-1707	9,851,867	326,565	2018	5 × 10^-6^	38.1	http://app.mrbase.org
Tea intake	ukb-b-6066	9,851,867	447,485	2018	5 × 10^-8^	25.5	http://app.mrbase.org
Unsalted nuts intake	ukb-b-12217	9,851,867	64,949	2018	5 × 10^-6^	23.1	http://app.mrbase.org
Unsalted peanuts intake	ukb-b-15555	9,851,867	64,949	2018	5 × 10^-6^	67.7	http://app.mrbase.org
Yogurt intake	ukb-b-7753	9,851,867	64,949	2018	5 × 10^-6^	24.4	http://app.mrbase.org
Never eat eggs, dairy, wheat, sugar: Dairy products	ukb-b-18909	9,851,867	461,046	2018	5 × 10^-6^	22.3	http://app.mrbase.org
Never eat eggs, dairy, wheat, sugar: Eggs or foods containing eggs	ukb-b-17455	9,851,867	461,046	2018	5 × 10^-6^	23.1	http://app.mrbase.org
Never eat eggs, dairy, wheat, sugar: I eat all of the above	ukb-b-2393	9,851,867	461,046	2018	5 × 10^-8^	26.0	http://app.mrbase.org
Never eat eggs, dairy, wheat, sugar: Sugar or foods/drinks containing sugar	ukb-b-5495	9,851,867	461,046	2018	5 × 10^-8^	29.1	http://app.mrbase.org
Never eat eggs, dairy, wheat, sugar: Wheat products	ukb-b-3599	9,851,867	461,046	2018	5 × 10^-8^	20.5	http://app.mrbase.org
Male infertility	finn-b-N14_MALEINFERT	16,377,329	73,479	2021	NA	NA	https://www.finngen.fi/en
Female infertility	finn-b-N14_FEMALEINFERT	16,377,038	75,450	2021	NA	NA	https://www.finngen.fi/en

NA, Not available.

**Figure 2:**
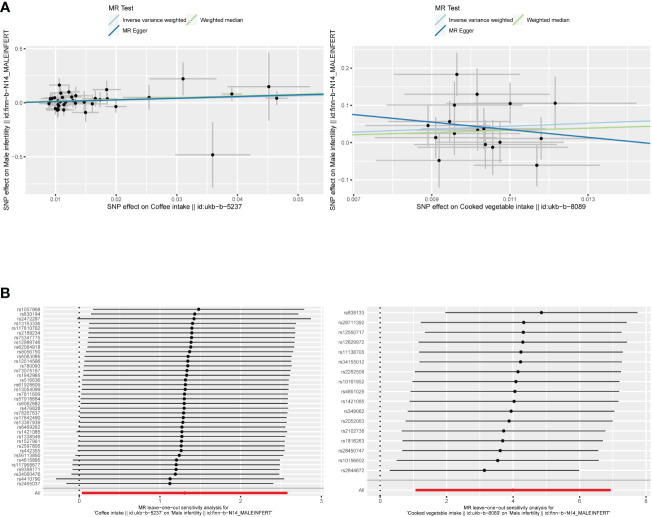
**(A)** Scatter plots of the causal relationship between coffee and cooked vegetable intakes, and male infertility. **(B)** The leave-one-out plots of the causal relationship between coffee and cooked vegetable intakes, and male infertility.

**Figure 3:**
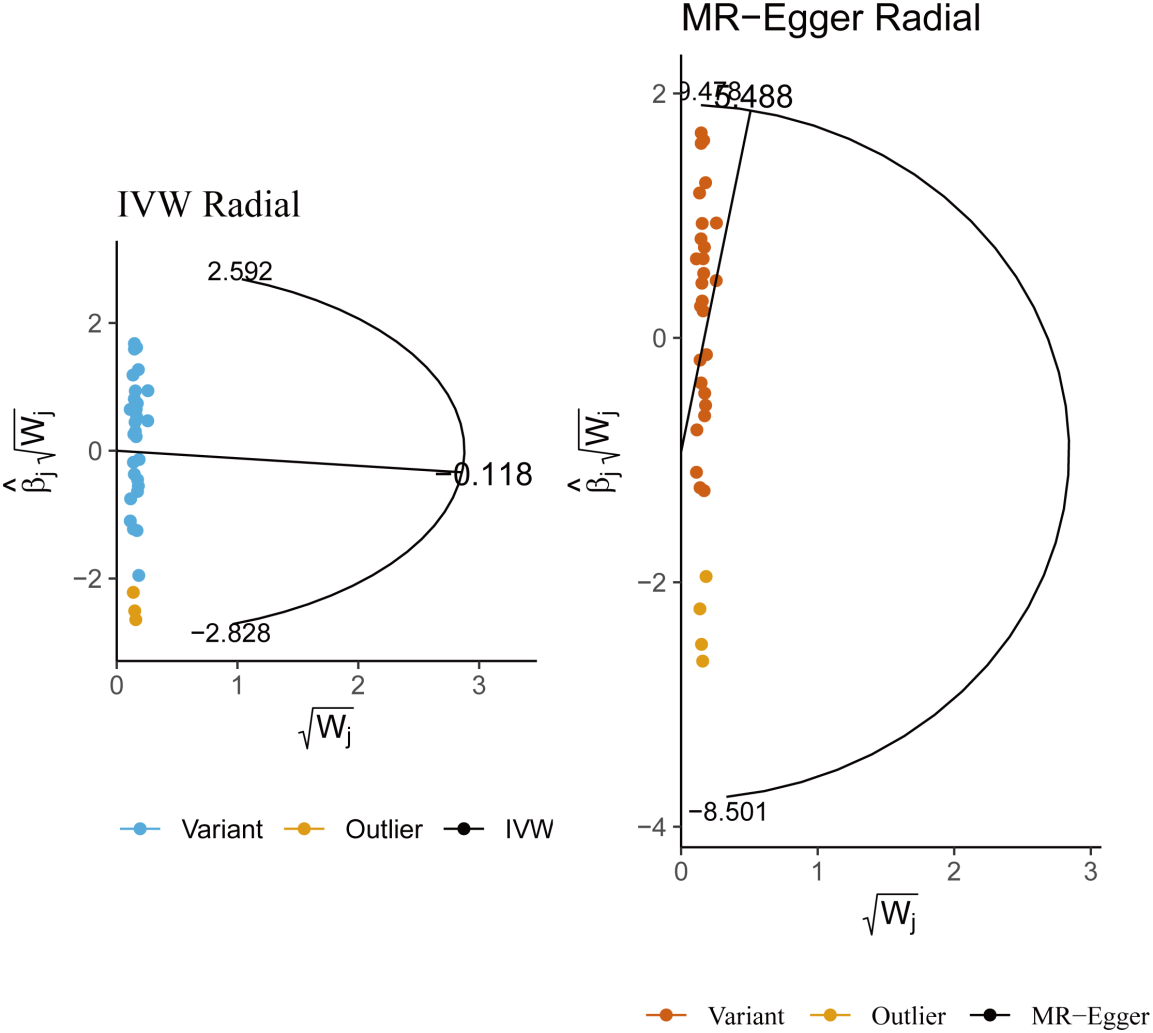
Radial Mendelian randomization analysis of the causal relationship between lamb intake and male infertility.

In sensitivity analysis, neither the IVW test nor the MR-Egger test showed any heterogeneity in the results of causality between all diets and male infertility (**Supplementary Table 2**). Additionally, the MR-Egger intercept test did not detect horizontal pleiotropy of the causality results, except for the relationship between processed meat intake and male infertility (P = 0.0262). The MR-PRESSO and radial MR tests found pleiotropy in lamb intake (P = 0.0460) alone (**Figure 3**, **Table 2**), and no significant outliers. Based on the leave-one-out analysis, no single SNP had a large impact on the robustness of the results after the individual removal tests (**Figure 2B**).

**Table 2:** Radial MR method to test for heterogeneity and horizontal pleiotropy in IVW and MR Egger.

		Estimate	SE	t value	P value
Lamb intake and male infertility	Radial IVW				
	Effect (1st)	-0.1180	1.3269	-0.0889	0.9290
	Iterative	-0.1180	1.3269	-0.0889	0.9290
	Exact (FE)	-0.1223	1.1002	-0.1111	0.9114
	Exact (RE)	-0.1209	1.2532	-0.0965	0.9237
	Q-Statistic for heterogeneity	NA	NA	NA	0.0514
	Radial MR-Egger				
	Intercept	-0.9330	1.1165	-0.8356	0.4101
	Wj	5.4881	6.8400	0.8023	0.4288
	Q-Statistic for heterogeneity	NA	NA	NA	0.0494
Coffee intake and female infertility	Radial IVW				
	Effect (1st)	-0.3124	0.2746	-1.1374	0.2553
	Iterative	-0.3124	0.2746	-1.1375	0.2553
	Exact (FE)	-0.3190	0.2219	-1.4376	0.1505
	Exact (RE)	-0.3167	0.2569	-1.2326	0.2254
	Q-Statistic for heterogeneity	NA	NA	NA	0.0200
	Radial MR-Egger				
	Intercept	-0.0769	0.4145	-0.1856	0.8537
	Wj	-0.2207	0.5669	-0.3893	0.6993
	Q-Statistic for heterogeneity	NA	NA	NA	0.0155
Red wine intake and female infertility	Radial IVW				
	Effect (1st)	-0.8267	0.5624	-1.4698	0.1416
	Iterative	-0.8274	0.5627	-1.4703	0.1414
	Exact (FE)	-0.8732	0.3559	-2.4529	0.0141
	Exact (RE)	-0.8446	0.5453	-1.5490	0.1397
	Q-Statistic for heterogeneity	NA	NA	NA	0.0005
	Radial MR-Egger				
	Intercept	-0.5373	2.1516	-0.2497	0.8059
	Wj	-0.0316	3.2357	-0.0097	0.9923
	Q-Statistic for heterogeneity	NA	NA	NA	0.0003

NA, Not available.

### Diet and infertility in women

**Figure 4:**
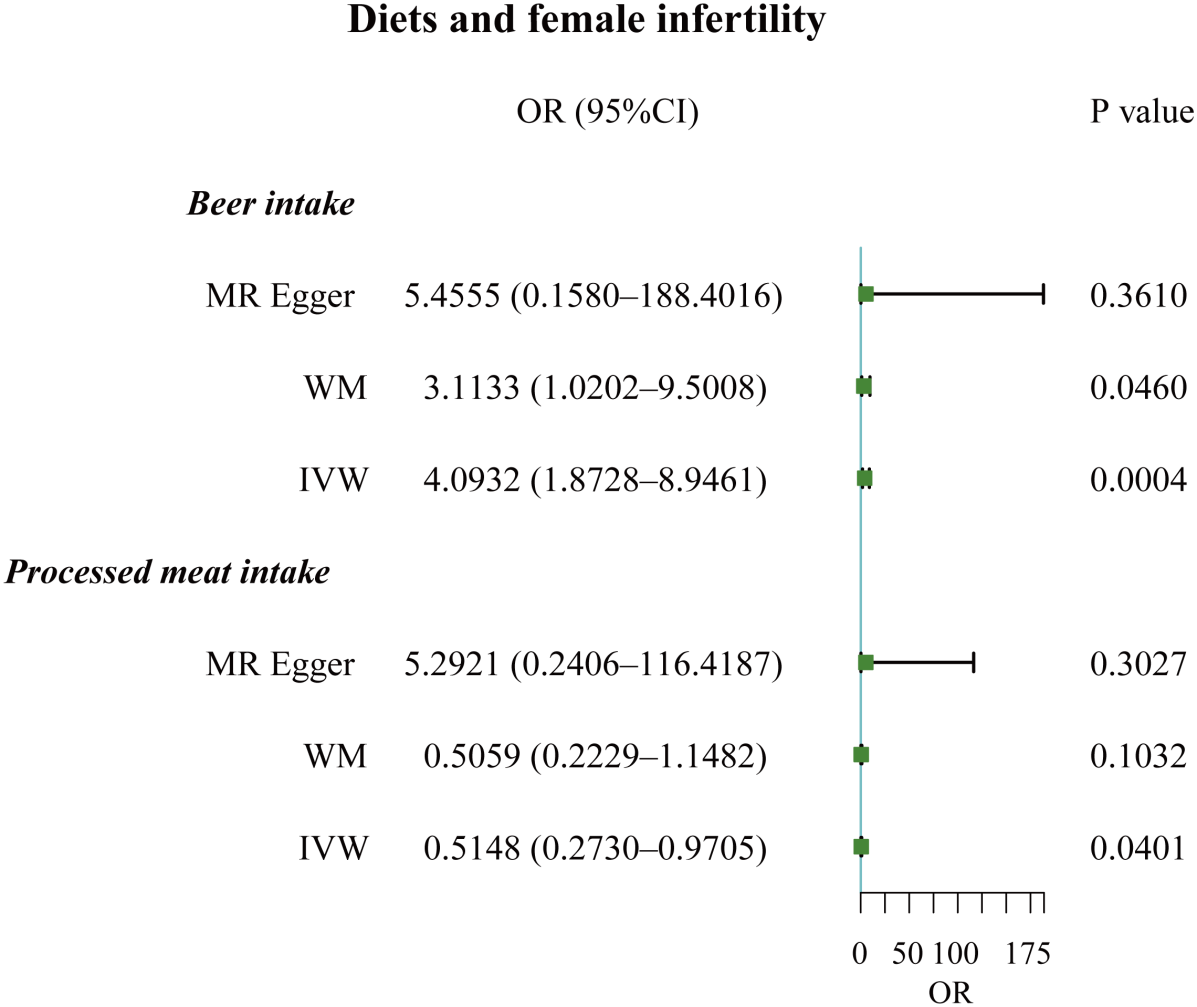
Forest plot of the causal associations between beer and processed meat intakes, and female infertility.

**Figure 5:**
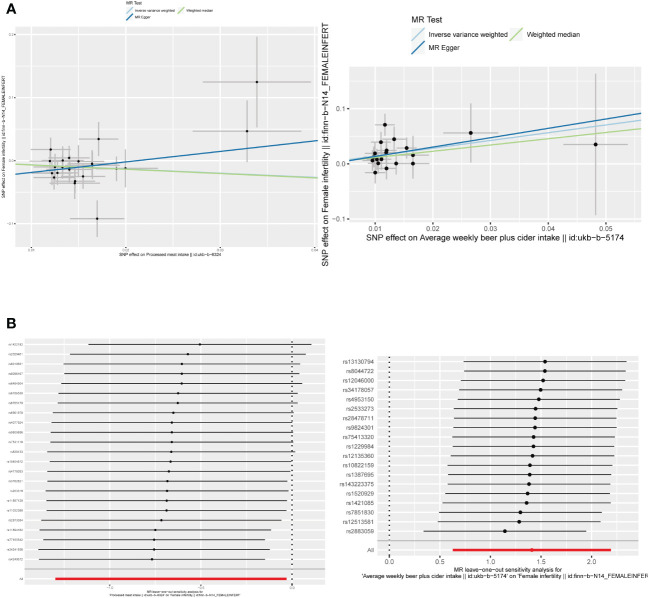
**(A)** Scatter plots of the causal relationship between beer and processed meat intakes, and female infertility. **(B)** The leave-one-out plots of the causal relationship between beer and processed meat intakes, and female infertility.

The F-statistic values for all IVs were higher than 10, avoiding the risk of weak instrumental bias (**Table 1**). TSMR analysis revealed a negative association between processed meat intake and female infertility, suggesting that processed meat reduced the risk of female infertility (OR, 0.5148; 95% CI, 0.2730 – 0.9705; P = 0.0401). Additionally, beer has been found to be a risk factor for female infertility (OR, 4.0932; 95% CI, 1.8728 – 8.9461; P = 0.0004) (**Figures 4**, **5A**; **Supplementary Table 3**).

**Figure 6:**
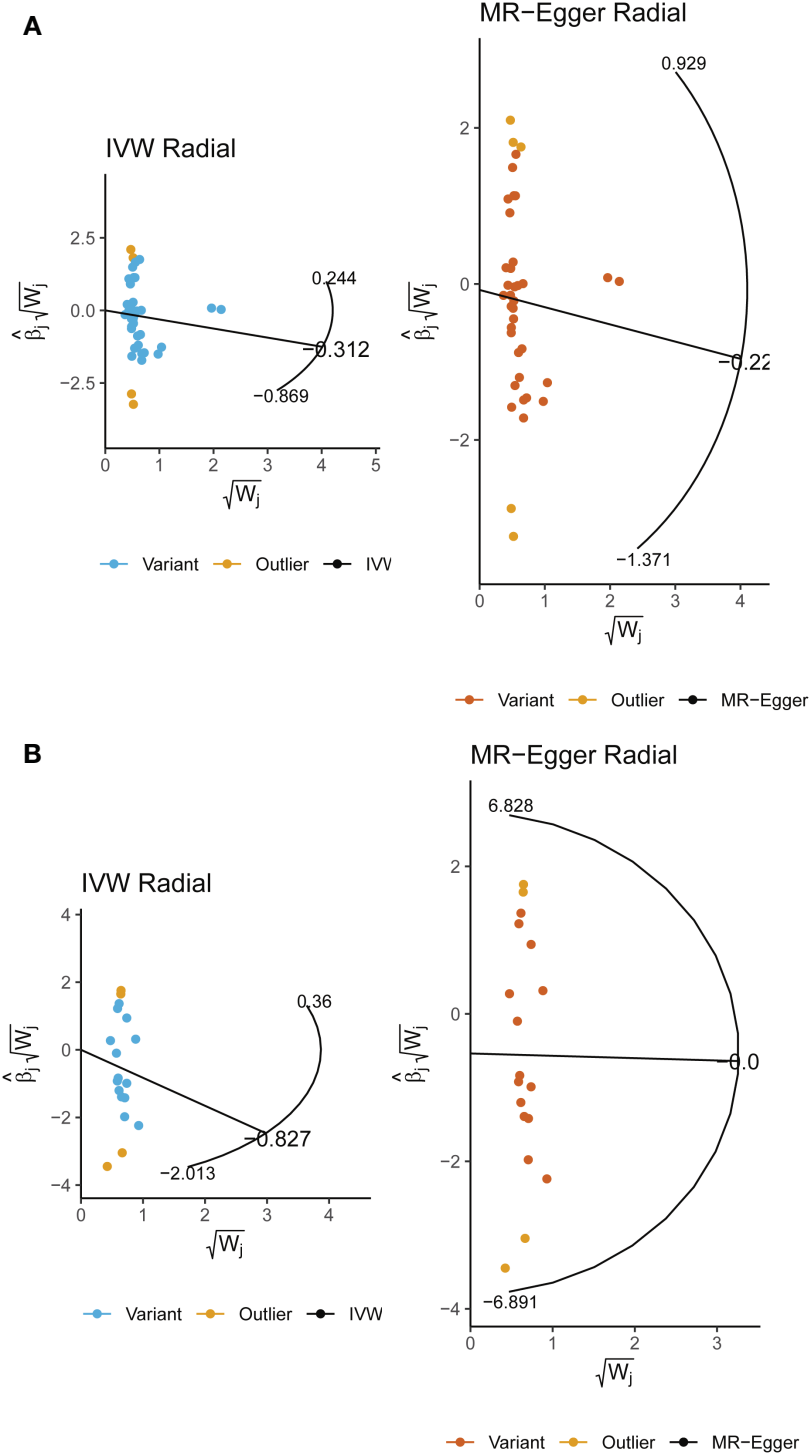
**(A)** Radial Mendelian randomization analysis of the causal relationship between coffee intake and female infertility. **(B)** Radial Mendelian randomization analysis of the causal relationship between red wine intake and female infertility.

By employing Cochran’s Q test, we found evidence of heterogeneity in bread, cereal, coffee, salted peanuts, and red wine intakes. No horizontal pleiotropy was detected in the MR-Egger intercept test (**Supplementary Table 4**). Additionally, radial MR and MR-PRESSO global test found outliers in coffee and red wine intakes (**Figure 6**, **Table 2**). After excluding outliers (rs13163336 for coffee intake; rs62573521 for red wine intake), the original causality did not change (**Supplementary Table 5**). Based on the leave-one-out analysis, no single SNP had a large impact on the robustness of the results after the individual removal tests (**Figure 5B**).

### Multivariate mendelian randomization

To examine whether diet impacts infertility by working through other factors, we performed MVMR analyses (**Supplementary Table 6**). After adjusting for zinc and selenium, cooked vegetable intake remained as having a significant negative effect on male infertility (OR, 34.3751; 95% CI, 2.1094 – 560.1708; P = 0.0130). For women, we found that processed meat (OR, 0.3718; 95% CI, 0.1876 – 0.7367; P = 0.0046) and selenium (OR, 7.4474e-12; 95% CI, 5.4780e-22 – 1.0125e-01; P = 0.0314) reduced the risk of female infertility after adjustments. When adjusted for monounsaturated fatty acids, polyunsaturated fatty acids, and saturated fatty acids, processed meat intake still displayed a protective effect against female infertility (OR, 0.4875; 95% CI, 0.2600 – 0.9142; P = 0.0251).

## Discussion

The present study investigated the impact of diet on male and female infertility using TSMR, MVMR, and radial MR methods. Our data showed that coffee and cooked vegetables increased the risk of male infertility while beer intake positively associated with female infertility. Processed meat intake was found to be protective against female infertility. Additionally, selenium may improve female fertility.

Reproduction, nutrition, and energy metabolism are closely linked and regulate each other (17). Nutritional imbalance such as excessive calorie intake and malnutrition, coupled with lack of exercise, can cause an accumulation of body fat that in turn may lead to non-communicable diseases such as diabetes, cancer, and cardiovascular disease (18, 19). These lifestyle factors can negatively impact male fertility by decreasing sperm quality, including semen volume and sperm motility (20). For women, an unhealthy diet can negatively affect menstruation, fertility, and maternal and fetal health (21).

Coffee is widely consumed around the world, and whether its effects on fertility are large or small, it may have serious public health implications. Current research disputes an impact of coffee or caffeine on fertility. A systematic review involving 19,967 men found that semen parameters were unaffected by caffeine intake, primarily from coffee, tea, and cocoa beverages, in most studies (22). Another study supported this idea by observing no association between moderate caffeine (≤800mg/d) or cola (≤1L/d) intake and decreased semen quality (23). However, Belloc et al. found that nearly 76% of caffeine consumers (3.0 ± 1.8 cups of coffee/day) noticed a slight increase in semen volume; and sperm viability was higher in men of childbearing age who drank six or more cups of coffee per day (24, 25). Most of the current research has focused solely on the effects of caffeine on fertility, but caffeine is widely available in tea, cola, and cocoa drinks that contain many other ingredients besides caffeine (26, 27). This means that it is difficult to separate spurious associations from potential causal associations, and that caffeine may not be the only factor in the effect of these beverages on fertility. Our study avoided confounders as much as possible and revealed the potentially harmful effects of coffee, but not caffeine, on male infertility. The mechanism by which coffee intake impairs male reproductive function is related to double-stranded DNA breaks and sperm aneuploidy, but not DNA adducts (28).

Vegetables are high in water content, certain minerals (like magnesium and potassium) that have antioxidant qualities, fiber, folate, and antioxidant-rich vitamins (like Vitamin A, Vitamin C, polyphenols, and β-carotene). Vegetable intake is known to be a reproductively beneficial part of diet. A cross-sectional study has confirmed that elevated vegetable consumption is associated with higher sperm concentration, motility, and total and progressive semen motility (29). In another case-control study, total vegetable intake was associated with a lower risk of idiopathic asthenozoospermia (30). Vegetables are rich in antioxidants that are used to reverse the negative effects of high reactive oxygen species (ROS) concentrations on sperm viability, vitality and concentration, as well as on miscarriages and abnormalities during offspring development (31). Moreover, folate that is found primarily in green leafy vegetables plays an important role in DNA maintenance as well as transfer RNA and protein synthesis. Given that DNA synthesis is an important component of spermatogenesis, it is likely that folic acid affects this process (32). However, few studies have distinguished between the effects of different cooking methods on vegetable efficacy. In the previously reported cooking-affected relationships between vegetables and noncommunicable diseases, for example, the causality of some vegetables and blood pressure can change with cooking style (33). Also, of the 11 studies on raw and cooked vegetables, nine showed a statistically significant negative association between these cancers and raw vegetable intake, but only four reports showed a negative association with cooked vegetables (34). These differences may stem from changes in food structure and nutrient availability, and a disruption of digestive enzymes (34). Our results demonstrated that an increased cooked vegetable intake resulted in infertility, and in conjunction with another similar study, that raw vegetables maintained or improved semen quality (35). Advising couples trying to conceive or people with infertility to use more raw vegetables appears to be beneficial.

A case-control study showed an increase in infertility due to ovulatory factors or endometriosis and moderate to high levels of alcohol use (36). In another prospective study, alcohol intake was associated with reduced fertility, even among women who drank five or fewer drinks per week (37). A recent dose-response meta-analysis confirmed this idea, with a linear association between reduced fertility and each 12.5 g/day increase in alcohol consumption (38). Many past publications have revealed an association between alcohol consumption and fertility; however, the results are largely controversial (39, 40). This inconsistency may stem from the type of alcohol consumption, the dose of alcohol consumed, sample characteristics, and study design. Furthermore, alcohol consumption has been found to negatively affect sperm quality (41–43). Heavy drinkers have increased rates of chromatin de-condensation and sperm DNA fragmentation (44, 45). It is also important to emphasize the effects of alcohol consumption on the offspring, as women who consume alcohol during pregnancy cause the immature fetal brain exposed to ethanol to experience massive neuronal death, resulting in fetal alcohol spectrum disorders that are one of the leading causes of intellectual disability in western countries (46). Paternal alcohol consumption not only produces severe brain alterations in their offspring but is also associated with restricted reproductive development in the offspring and reduced placental efficiency (47–49).

Despite the inevitable loss of nutrients (such as zinc) from meat because of the cooking process, as well as the possible formation of unfavorable heterocyclic amines during processing leading to cell damage and loss of biological function, resulting in processed meat being reported to have adverse effects on male reproduction (50, 51). However, we found that processed meat may have a protective effect against female infertility. It is possible that as consumer demand for the health benefits of food has increased, the processed meat industry has developed low-fat, healthy meat products reformulated by substituting vegetable oils for animal fats, and that this trend has led to a modest improvement in human fertility (52). Moreover, we have demonstrated the application of zinc in promoting reproductive health, while meat, especially liver and kidney, is particularly high in selenium; this may also explain the reproductive benefits of processed meat (53).

The role of subjective confounding factors in the effect of diet on infertility is still not negligible. Inadequate diets, whether low calorie or unhealthy excess calorie intake, negatively affects physiological reproductive function and greatly increases the risk of infertility (54–56). High body mass index (BMI) interferes with reproductive potential. Men with obesity have decreased total and bioavailable testosterone levels (57); and central, peripheral, and testicular factors affect the hypothalamic-pituitary-gonadal (HPG) axis, contributing to the secondary hypogonadism, which may lead to reduced sexual desire, erectile dysfunction, and impaired spermatogenesis (57). In women, excess adipose tissue leads to negative feedback on the hypothalamic-pituitary-ovarian (HPO) axis and affects gonadotropin production (58). This manifests as menstrual abnormalities and ovulatory dysfunction (59). Moreover, polycystic ovary syndrome (PCOS) is a common endocrine disorder in women, and patients are usually overweight and have menstrual irregularities. And the triad of obesity, infertility and PCOS is reported to be inextricably linked, and its prevalence is rising (60). Obesity can lead to insulin resistance that exacerbates the symptoms of PCOS, in that the adverse effects of PCOS are more prevalent in obese women (61). Additionally, insulin resistance contributes to ovulation disorders and abnormal endometrial structure (62, 63). As discussed above, factors such as metabolic disorders and hormonal imbalancement still play a role in the effects of diet on infertility, suggesting that the use of causal language is necessary when discussing the link between diet and infertility. Although the results of the analysis based on the three assumptions of MR are stable and reliable, given the variety of confounders with large sample sizes, we do not have assessment parameters available for these factors. Therefore, it is essential for clinicians to consider the triad of diet, the above factors and infertility when developing prevention or treatment options. This also suggests that individualized diets should be tailored to the patients, rather than specific recipes. Our study has some limitations. First, the participants of the GWAS data we utilized were predominantly from Europe, and the applicability of the causal relationships in the study to populations in other regions still requires further validation. Second, the level of food intake is not known, and the amount of intake may bias the results. Finally, not all participants were of childbearing age, and the effect of age on fertility may outweigh dietary effects. Our study revealed the impact of diet on infertility in men and women and provided clinicians with an inexpensive and concise option for guiding couples of childbearing age to prevent infertility. In the future, we hope to design better prospective studies with larger sample sizes to validate our conclusions.

## Conclusion

We utilized TSMR and MVMR methods to explore the effect of diet on infertility. We found that coffee and cooked vegetable intakes increased the risk of male infertility. And there was a positive correlation between beer intake and female infertility. But processed meat was negatively associated with female infertility. Moreover, MVMR found selenium to be a protective factor for female infertility. We expect prospective studies with larger samples to justify our conclusions in the future.

## Data availability statement

The datasets presented in this study can be found in online repositories. The names of the repository/repositories and accession number(s) can be found in the article/**Supplementary Material**.

## Ethics statement

Ethical approval was not required for the study involving humans in accordance with the local legislation and institutional requirements. Written informed consent to participate in this study was not required from the participants or the participants’ legal guardians/next of kin in accordance with the national legislation and the institutional requirements.

## Author contributions

XC: Data curation, Formal analysis, Methodology, Resources, Software, Visualization, Writing – original draft, Writing – review & editing. CR: Software, Writing – original draft. CW: Methodology, Writing – original draft. XL: Methodology, Writing – original draft, Writing – review & editing.
