# Supplementary material for: Mendelian randomization reveals the impact of diet on infertility in men and women
Source: Front Endocrinol (Lausanne). 2024 Apr 23;15:1376800. doi: 10.3389/fendo.2024.1376800 (PMC11074355; doi:10.3389/fendo.2024.1376800)
Supplement: Supplementary file 1 [file DataSheet_1.docx]

***Supplementary Material***

**Supplementary Table 1.** Mendelian randomization analysis of causal relationship between diets and male infertility.

**Supplementary Table 2.** Heterogeneity and pleiotropy between diets and male infertility assessed using different methods.

**Supplementary Table 3.** Mendelian randomization analysis of causal relationship between diets and female infertility.

**Supplementary Table 4.** Heterogeneity and pleiotropy between diets and female infertility assessed using different methods.

**Supplementary Table 5.** The results of Mendelian randomization analysis after detection and exclusion of outliers by the MR-PRESSO test.

**Supplementary Table 6.** The results of Multivariate Mendelian randomization analysis.

**Supplementary Table 1.** Mendelian randomization analysis of causal relationship between diets and male infertility.

| Exposure | Outcome | SNPs | Methods | OR (95% CI) | P value |
| --- | --- | --- | --- | --- | --- |
| Bacon intake | Male infertility | 12 | MR Egger | 0.4438(0.0079–24.7165) | 0.7004 |
|  |  |  | Weighted median | 1.8940(0.2192–16.3598) | 0.5614 |
|  |  |  | Inverse variance weighted | 1.0845(0.2165–5.4328) | 0.9213 |
| Beef intake | Male infertility | 17 | MR Egger | 8.3887(8.1050e-07–8.6823e+07) | 0.8007 |
|  |  |  | Weighted median | 2.5653(6.9691e-02–9.4430e+01) | 0.6085 |
|  |  |  | Inverse variance weighted | 1.7550(1.2088e-01–2.5480e+01) | 0.6802 |
| Beer intake | Male infertility | 21 | MR Egger | 0.8629(8.7946e-06–84670.8325) | 0.9802 |
|  |  |  | Weighted median | 1.4543(5.7829e-02–36.5750) | 0.8199 |
|  |  |  | Inverse variance weighted | 0.6097(5.1678e-02–7.1941) | 0.6943 |
| Bread intake | Male infertility | 31 | MR Egger | 0.0002(4.5569e-08–1.0169) | 0.0617 |
|  |  |  | Weighted median | 0.7188(7.2574e-02–7.1201) | 0.7778 |
|  |  |  | Inverse variance weighted | 0.9695(1.4637e-01–6.4221) | 0.9744 |
| Cereal intake | Male infertility | 43 | MR Egger | 171.0580(0.3532–82832.3955) | 0.1115 |
|  |  |  | Weighted median | 0.3743(0.0432–3.2425) | 0.3723 |
|  |  |  | Inverse variance weighted | 1.3545(0.3129–5.8627) | 0.6847 |
| Cheese intake | Male infertility | 63 | MR Egger | 0.4911(0.0056–42.6828) | 0.7560 |
|  |  |  | Weighted median | 0.2881(0.0670–1.2386) | 0.0944 |
|  |  |  | Inverse variance weighted | 0.4727(0.1657–1.3485) | 0.1612 |
| Coffee intake | Male infertility | 40 | MR Egger | 4.7480(0.3668–61.4543) | 0.2409 |
|  |  |  | Weighted median | 4.8692(0.7662–30.9419) | 0.0934 |
|  |  |  | Inverse variance weighted | **3.6967(1.0348–13.2065)** | **0.0442** |
| Cooked vegetable intake | Male infertility | 17 | MR Egger | 3.6837e-05(2.8819e-19–4.7086e+09) | 0.5471 |
|  |  |  | Weighted median | 1.9655e+01(4.3359e-01–8.9093e+02) | 0.1259 |
|  |  |  | Inverse variance weighted | **54.7865(2.9011–1030.5500)** | **0.0076** |
| Dried fruit intake | Male infertility | 43 | MR Egger | 0.2537(0.0002–244.3569) | 0.6977 |
|  |  |  | Weighted median | 0.2040(0.0228–1.8185) | 0.1543 |
|  |  |  | Inverse variance weighted | 0.3303(0.0713–1.5293) | 0.1565 |
| Fresh fruit intake | Male infertility | 55 | MR Egger | 1.7623(3.0119e-03–1031.1969) | 0.8623 |
|  |  |  | Weighted median | 2.1353(1.4207e-01–32.0928) | 0.5832 |
|  |  |  | Inverse variance weighted | 1.7851(2.8242e-01–11.2832) | 0.5378 |
| Lamb intake | Male infertility | 32 | MR Egger | 0.1696(2.2633e-06–12721.4785) | 0.7589 |
|  |  |  | Weighted median | 6.1995(2.8008e-01–137.2236) | 0.2482 |
|  |  |  | Inverse variance weighted | 0.8886(6.5953e-02–11.9728) | 0.9290 |
| Milk intake | Male infertility | 20 | MR Egger | 1.9038(0.0279–129.5003) | 0.7683 |
|  |  |  | Weighted median | 1.4476(0.0891–23.4932) | 0.7947 |
|  |  |  | Inverse variance weighted | 1.5422(0.2181–10.9035) | 0.6641 |
| Non-oily fish intake | Male infertility | 11 | MR Egger | 6.9628e-05(2.8913e-11–167.6760) | 0.2336 |
|  |  |  | Weighted median | 3.5408e-01(4.9068e-03–25.5511) | 0.6343 |
|  |  |  | Inverse variance weighted | 3.2924e-01(1.5115e-02–7.1717) | 0.4797 |
| Oily fish intake | Male infertility | 64 | MR Egger | 1.4249(0.0104–193.3827) | 0.8880 |
|  |  |  | Weighted median | 0.7794(0.1551–3.9158) | 0.7622 |
|  |  |  | Inverse variance weighted | 0.4986(0.1576–1.5768) | 0.2361 |
| Pork intake | Male infertility | 13 | MR Egger | 113.0704(5.9586e-08–2.1455e+11) | 0.6728 |
|  |  |  | Weighted median | 1.0862(9.6239e-03–1.2259e+02) | 0.9726 |
|  |  |  | Inverse variance weighted | 0.8353(3.0452e-02–2.2916e+01) | 0.9152 |
| Poultry intake | Male infertility | 8 | MR Egger | 1.4075e+39(9.9121e-19–1.9988e+96) | 0.2371 |
|  |  |  | Weighted median | 1.6666e+01(5.8225e-02–4.7706e+03) | 0.3296 |
|  |  |  | Inverse variance weighted | 3.1117(2.9668e-02–3.2636e+02) | 0.6325 |
| Processed meat intake | Male infertility | 23 | MR Egger | 1.0201e-05(1.5741e-09–0.0661) | 0.0179 |
|  |  |  | Weighted median | 3.4060e-01(2.9557e-02–3.9250) | 0.3878 |
|  |  |  | Inverse variance weighted | 3.6753e-01(6.1731e-02–2.1882) | 0.2714 |
| Raw vegetable intake | Male infertility | 24 | MR Egger | 0.0026(1.2831e-09–5405.5296) | 0.4342 |
|  |  |  | Weighted median | 0.3177(4.1913e-03–24.0910) | 0.6036 |
|  |  |  | Inverse variance weighted | 0.4349(2.0480e-02–9.2391) | 0.5934 |
| Red wine intake | Male infertility | 19 | MR Egger | 17.1700(0.0001–1.8309e+06) | 0.6367 |
|  |  |  | Weighted median | 2.5207(0.1287–4.9358e+01) | 0.5423 |
|  |  |  | Inverse variance weighted | 4.1942(0.4236–4.1520e+01) | 0.2202 |
| Salted nuts intake | Male infertility | 23 | MR Egger | 0.0873(0.0021–3.5060) | 0.2096 |
|  |  |  | Weighted median | 0.3435(0.0180–6.5263) | 0.4769 |
|  |  |  | Inverse variance weighted | 0.3780(0.0406–3.5142) | 0.3924 |
| Salted peanuts intake | Male infertility | 14 | MR Egger | 163.4639(0.8950–29854.1245) | 0.0813 |
|  |  |  | Weighted median | 2.3157(0.0522–102.5607) | 0.6641 |
|  |  |  | Inverse variance weighted | 2.0199(0.1251– 32.5960) | 0.6202 |
| Spirits intake | Male infertility | 46 | MR Egger | 0.5231(0.0186–14.6645) | 0.7051 |
|  |  |  | Weighted median | 0.6307(0.0862–4.6146) | 0.6499 |
|  |  |  | Inverse variance weighted | 1.3927(0.3347–5.7943) | 0.6487 |
| Tea intake | Male infertility | 41 | MR Egger | 3.3985(0.2724–42.3894) | 0.3480 |
|  |  |  | Weighted median | 2.0809(0.3485–12.4252) | 0.4215 |
|  |  |  | Inverse variance weighted | 0.6524(0.2034–2.0922) | 0.4726 |
| Unsalted nuts intake | Male infertility | 15 | MR Egger | 6.8094(0.0125–3682.7371) | 0.5604 |
|  |  |  | Weighted median | 1.4436(0.0419–49.6948) | 0.8388 |
|  |  |  | Inverse variance weighted | 0.6603(0.0574–7.5965) | 0.7391 |
| Unsalted peanuts intake | Male infertility | 45 | MR Egger | 0.0907(0.0002–27.8322) | 0.4159 |
|  |  |  | Weighted median | 0.3091(0.0043–21.7584) | 0.5886 |
|  |  |  | Inverse variance weighted | 1.5105(0.0862–26.4575) | 0.7776 |
| Yogurt intake | Male infertility | 10 | MR Egger | 24.5953(0.1381–4379.1980) | 0.2650 |
|  |  |  | Weighted median | 0.1259(0.0138–1.1482) | 0.0661 |
|  |  |  | Inverse variance weighted | 0.6045(0.0971– 3.7631) | 0.5896 |
| Never eat eggs, dairy, wheat, sugar: Dairy products | Male infertility | 5 | MR Egger | 3.8997e+26(1.5925e-27–9.5493e+79) | 0.4009 |
|  |  |  | Weighted median | 7.4260e+09(1.2867e-08–4.2856e+27) | 0.2760 |
|  |  |  | Inverse variance weighted | 1.5003e+08(8.0938e-10–2.7812e+25) | 0.3533 |
| Never eat eggs, dairy, wheat, sugar: Eggs or foods containing eggs | Male infertility | 8 | MR Egger | 6.6258e+08(8.1348e-31–5.3967e+47) | 0.6723 |
|  |  |  | Weighted median | 4.4021e-03(2.1116e-17–9.1771e+11) | 0.7470 |
|  |  |  | Inverse variance weighted | 1.8645e-03(1.7272e-14–2.0126e+08) | 0.6277 |
| Never eat eggs, dairy, wheat, sugar: I eat all of the above | Male infertility | 28 | MR Egger | 184.325(6.6647e-11–5.0978e+14) | 0.7244 |
|  |  |  | Weighted median | 33.2208(3.3284e-02–3.3157e+04) | 0.3200 |
|  |  |  | Inverse variance weighted | 4.1345(2.1570e-02–7.9246e+02) | 0.5965 |
| Never eat eggs, dairy, wheat, sugar: Sugar or foods/drinks containing sugar | Male infertility | 22 | MR Egger | 1.2389e-08(1.2559e-27–1.2222e+11) | 0.4246 |
|  |  |  | Weighted median | 2.9971e+01(2.2318e-02–4.0248e+04) | 0.3548 |
|  |  |  | Inverse variance weighted | 5.0008(1.8306e-02–1.3660e+03) | 0.5738 |
| Never eat eggs, dairy, wheat, sugar: Wheat products | Male infertility | 7 | MR Egger | 0.8563(4.2181e-12–1.7385e+11) | 0.9911 |
|  |  |  | Weighted median | 21.2791(1.0633e-05–4.2581e+07) | 0.6795 |
|  |  |  | Inverse variance weighted | 0.2273(2.7156e-08–1.9026e+06) | 0.8554 |

**Supplementary Table 2.** Heterogeneity and pleiotropy between diets and male infertility assessed using different methods.

| Exposure | Outcome | Methods | Heterogeneity | | Pleiotropy | | | MR-PRESSO |
| --- | --- | --- | --- | --- | --- | --- | --- | --- |
|  |  |  | Q | P value | Egger_intercept | SE | P value | P value |
| Bacon intake | Male infertility | MR Egger | 9.0433 | 0.5279 | 0.0340 | 0.0716 | 0.6446 | 0.5920 |
|  |  | IVW | 9.2694 | 0.5970 |  |  |  |  |
| Beef intake | Male infertility | MR Egger | 9.5292 | 0.6571 | -0.0198 | 0.1033 | 0.8505 | 0.8230 |
|  |  | IVW | 9.5662 | 0.7289 |  |  |  |  |
| Beer intake | Male infertility | MR Egger | 20.8572 | 0.2327 | -0.0043 | 0.0713 | 0.9522 | 0.3830 |
|  |  | IVW | 20.8617 | 0.2864 |  |  |  |  |
| Bread intake | Male infertility | MR Egger | 29.7503 | 0.2337 | 0.1212 | 0.0607 | 0.0571 | 0.0970 |
|  |  | IVW | 34.4825 | 0.1233 |  |  |  |  |
| Cereal intake | Male infertility | MR Egger | 38.2157 | 0.4140 | -0.0706 | 0.0447 | 0.1232 | 0.3740 |
|  |  | IVW | 40.7848 | 0.3489 |  |  |  |  |
| Cheese intake | Male infertility | MR Egger | 66.3856 | 0.2375 | -0.0006 | 0.0380 | 0.9862 | 0.3180 |
|  |  | IVW | 66.3860 | 0.2663 |  |  |  |  |
| Coffee intake | Male infertility | MR Egger | 29.7951 | 0.7574 | -0.0047 | 0.0214 | 0.8264 | 0.8390 |
|  |  | IVW | 29.8439 | 0.7920 |  |  |  |  |
| Cooked vegetable intake | Male infertility | MR Egger | 17.1691 | 0.3088 | 0.1467 | 0.1704 | 0.4027 | 0.3580 |
|  |  | IVW | 18.0178 | 0.3228 |  |  |  |  |
| Dried fruit intake | Male infertility | MR Egger | 33.4647 | 0.7199 | 0.0033 | 0.0428 | 0.9388 | 0.8360 |
|  |  | IVW | 33.4707 | 0.7575 |  |  |  |  |
| Fresh fruit intake | Male infertility | MR Egger | 53.7366 | 0.3699 | 0.0001 | 0.0297 | 0.9967 | 0.3930 |
|  |  | IVW | 53.7366 | 0.4076 |  |  |  |  |
| Lamb intake | Male infertility | MR Egger | 43.5016 | 0.0408 | 0.0184 | 0.0618 | 0.7682 | 0.046 |
|  |  | IVW | 43.6343 | 0.0514 |  |  |  |  |
| Milk intake | Male infertility | MR Egger | 13.5197 | 0.7598 | -0.0045 | 0.0413 | 0.9133 | 0.8160 |
|  |  | IVW | 13.5319 | 0.8102 |  |  |  |  |
| Non-oily fish intake | Male infertility | MR Egger | 8.0231 | 0.5318 | 0.1050 | 0.0910 | 0.2781 | 0.5150 |
|  |  | IVW | 9.3555 | 0.4987 |  |  |  |  |
| Oily fish intake | Male infertility | MR Egger | 64.3233 | 0.2956 | -0.0155 | 0.0361 | 0.6678 | 0.3840 |
|  |  | IVW | 64.5261 | 0.3214 |  |  |  |  |
| Pork intake | Male infertility | MR Egger | 12.0537 | 0.3596 | -0.0509 | 0.1117 | 0.6571 | 0.4420 |
|  |  | IVW | 12.2817 | 0.4233 |  |  |  |  |
| Poultry intake | Male infertility | MR Egger | 5.8042 | 0.3257 | -0.9641 | 0.7269 | 0.2420 | 0.2490 |
|  |  | IVW | 7.8465 | 0.2495 |  |  |  |  |
| Processed meat intake | Male infertility | MR Egger | 16.8975 | 0.7172 | 0.1592 | 0.0666 | 0.0262 | 0.4610 |
|  |  | IVW | 22.6175 | 0.4235 |  |  |  |  |
| Raw vegetable intake | Male infertility | MR Egger | 13.8438 | 0.6781 | 0.0552 | 0.0784 | 0.4907 | 0.8670 |
|  |  | IVW | 14.3400 | 0.7066 |  |  |  |  |
| Red wine intake | Male infertility | MR Egger | 21.3821 | 0.1642 | -0.0204 | 0.0839 | 0.8105 | 0.2820 |
|  |  | IVW | 21.4615 | 0.2063 |  |  |  |  |
| Salted nuts intake | Male infertility | MR Egger | 23.6517 | 0.3102 | 0.0374 | 0.0383 | 0.3400 | 0.3700 |
|  |  | IVW | 24.7253 | 0.3103 |  |  |  |  |
| Salted peanuts intake | Male infertility | MR Egger | 9.0121 | 0.6207 | -0.0935 | 0.0483 | 0.0793 | 0.4690 |
|  |  | IVW | 12.7501 | 0.3874 |  |  |  |  |
| Spirits intake | Male infertility | MR Egger | 46.4831 | 0.2928 | 0.0160 | 0.0251 | 0.5269 | 0.3100 |
|  |  | IVW | 46.9334 | 0.3144 |  |  |  |  |
| Tea intake | Male infertility | MR Egger | 46.1512 | 0.1708 | -0.0350 | 0.0243 | 0.1580 | 0.1590 |
|  |  | IVW | 48.6691 | 0.1379 |  |  |  |  |
| Unsalted nuts intake | Male infertility | MR Egger | 12.2716 | 0.5055 | -0.0633 | 0.0803 | 0.4445 | 0.5120 |
|  |  | IVW | 12.8934 | 0.5349 |  |  |  |  |
| Unsalted peanuts intake | Male infertility | MR Egger | 33.5614 | 0.8485 | 0.0475 | 0.0428 | 0.2725 | 0.8180 |
|  |  | IVW | 34.7969 | 0.8381 |  |  |  |  |
| Yogurt intake | Male infertility | MR Egger | 8.3571 | 0.3021 | -0.1323 | 0.0891 | 0.1813 | 0.6340 |
|  |  | IVW | 10.9872 | 0.2024 |  |  |  |  |
| Never eat eggs, dairy, wheat, sugar: Dairy products | Male infertility | MR Egger | 5.9279 | 0.1151 | -0.0991 | 0.1376 | 0.5234 | 0.2060 |
|  |  | IVW | 6.9529 | 0.1383 |  |  |  |  |
| Never eat eggs, dairy, wheat, sugar: Eggs or foods containing eggs | Male infertility | MR Egger | 3.8388 | 0.6984 | -0.0585 | 0.0965 | 0.5662 | 0.7980 |
|  |  | IVW | 4.2069 | 0.7556 |  |  |  |  |
| Never eat eggs, dairy, wheat, sugar: I eat all of the above | Male infertility | MR Egger | 33.7311 | 0.0691 | -0.0226 | 0.0856 | 0.7937 | 0.1240 |
|  |  | IVW | 33.8337 | 0.0876 |  |  |  |  |
| Never eat eggs, dairy, wheat, sugar: Sugar or foods/drinks containing sugar | Male infertility | MR Egger | 24.6610 | 0.1720 | 0.1043 | 0.1164 | 0.3817 | 0.1850 |
|  |  | IVW | 25.7019 | 0.1758 |  |  |  |  |
| Never eat eggs, dairy, wheat, sugar: Wheat products | Male infertility | MR Egger | 8.8015 | 0.1172 | -0.0078 | 0.0584 | 0.8983 | 0.3650 |
|  |  | IVW | 8.8333 | 0.1831 |  |  |  |  |

**Supplementary Table 3.** Mendelian randomization analysis of causal relationship between diets and female infertility.

| Exposure | Outcome | SNPs | Methods | OR (95% CI) | P value |
| --- | --- | --- | --- | --- | --- |
| Bacon intake | Female infertility | 12 | MR Egger | 0.6002(0.1439–2.5029) | 0.4995 |
|  |  |  | Weighted median | 0.8658(0.3872–1.9357) | 0.7255 |
|  |  |  | Inverse variance weighted | 0.6973(0.4022–1.2090) | 0.1992 |
| Beef intake | Female infertility | 17 | MR Egger | 1.6294(0.0032–826.2519) | 0.8804 |
|  |  |  | Weighted median | 0.4740(0.1223–1.8375) | 0.2802 |
|  |  |  | Inverse variance weighted | 0.9192(0.3411–2.4768) | 0.8677 |
| Beer intake | Female infertility | 21 | MR Egger | 5.4555(0.1580–188.4016) | 0.3610 |
|  |  |  | Weighted median | 3.1133(1.0202–9.5008) | 0.0460 |
|  |  |  | Inverse variance weighted | **4.0932(1.8728–8.9461)** | **0.0004** |
| Bread intake | Female infertility | 31 | MR Egger | 6.6536(0.2507–176.5458) | 0.2679 |
|  |  |  | Weighted median | 1.4943(0.61796–3.6134) | 0.3726 |
|  |  |  | Inverse variance weighted | 0.7994(0.3966–1.6113) | 0.5313 |
| Cereal intake | Female infertility | 43 | MR Egger | 1.1033(0.0916–13.2864) | 0.9386 |
|  |  |  | Weighted median | 0.9794(0.4861–1.9731) | 0.9536 |
|  |  |  | Inverse variance weighted | 1.2619(0.7134–2.2321) | 0.4239 |
| Cheese intake | Female infertility | 63 | MR Egger | 3.0360(0.7228–12.7516) | 0.1346 |
|  |  |  | Weighted median | 0.8923(0.5453–1.4601) | 0.6501 |
|  |  |  | Inverse variance weighted | 0.8587(0.6113–1.2063) | 0.3798 |
| Coffee intake | Female infertility | 40 | MR Egger | 0.8519(0.2847–2.5483) | 0.7760 |
|  |  |  | Weighted median | 1.0117(0.5355–1.9113) | 0.9711 |
|  |  |  | Inverse variance weighted | 0.7316(0.4270–1.2534) | 0.2553 |
| Cooked vegetable intake | Female infertility | 17 | MR Egger | 0.0021(6.4177e-08–70.4846) | 0.2647 |
|  |  |  | Weighted median | 0.5802(1.5626e-01–2.1549) | 0.4162 |
|  |  |  | Inverse variance weighted | 0.9538(3.7006e-01–2.4586) | 0.9221 |
| Dried fruit intake | Female infertility | 43 | MR Egger | 3.3137(0.2475–44.3537) | 0.3709 |
|  |  |  | Weighted median | 0.8405(0.3898–1.8123) | 0.6576 |
|  |  |  | Inverse variance weighted | 0.6996(0.3911–1.2516) | 0.2287 |
| Fresh fruit intake | Female infertility | 55 | MR Egger | 0.6455(0.0626–6.6559) | 0.7146 |
|  |  |  | Weighted median | 0.7279(0.2871–1.8454) | 0.5034 |
|  |  |  | Inverse variance weighted | 0.9527(0.4842–1.8744) | 0.8884 |
| Lamb intake | Female infertility | 32 | MR Egger | 0.2535(0.0110–5.8029) | 0.3973 |
|  |  |  | Weighted median | 2.2901(0.7988–6.5657) | 0.1230 |
|  |  |  | Inverse variance weighted | 2.0830(0.9983–4.3464) | 0.0505 |
| Milk intake | Female infertility | 20 | MR Egger | 0.8182(0.1917–3.4921) | 0.7895 |
|  |  |  | Weighted median | 0.7803(0.3123–1.9490) | 0.5953 |
|  |  |  | Inverse variance weighted | 0.8272(0.4234–1.6162) | 0.5789 |
| Non-oily fish intake | Female infertility | 11 | MR Egger | 0.0299(0.0001–4.5090) | 0.2035 |
|  |  |  | Weighted median | 0.5254(0.1272–2.1691) | 0.3736 |
|  |  |  | Inverse variance weighted | 0.8645(0.3018–2.4760) | 0.7862 |
| Oily fish intake | Female infertility | 64 | MR Egger | 0.2533(0.0509–1.2595) | 0.0986 |
|  |  |  | Weighted median | 0.7999(0.4588–1.3943) | 0.4309 |
|  |  |  | Inverse variance weighted | 0.7161(0.4901–1.0462) | 0.0843 |
| Pork intake | Female infertility | 13 | MR Egger | 1.7731(0.0016–1922.1438) | 0.8752 |
|  |  |  | Weighted median | 1.6473(0.3861–7.0270) | 0.5000 |
|  |  |  | Inverse variance weighted | 1.8961(0.6190–5.8079) | 0.2625 |
| Poultry intake | Female infertility | 8 | MR Egger | 1.6129e+10(1.1067e-08–2.3507e+28) | 0.3208 |
|  |  |  | Weighted median | 4.0535(6.5146e-01–2.5221e+01) | 0.1334 |
|  |  |  | Inverse variance weighted | 2.9567(7.3416e-01–1.1907e+01) | 0.1272 |
| Processed meat intake | Female infertility | 23 | MR Egger | 5.2921(0.2406–116.4187) | 0.3027 |
|  |  |  | Weighted median | 0.5059(0.2229–1.1482) | 0.1032 |
|  |  |  | Inverse variance weighted | **0.5148(0.2730–0.9705)** | **0.0401** |
| Raw vegetable intake | Female infertility | 24 | MR Egger | 1.4185(0.0099–202.6562) | 0.8917 |
|  |  |  | Weighted median | 1.4820(0.3630–6.0502) | 0.5835 |
|  |  |  | Inverse variance weighted | 0.9444(0.3338–2.6718) | 0.9141 |
| Red wine intake | Female infertility | 19 | MR Egger | 0.0102(6.4460e-05–1.6454) | 0.0961 |
|  |  |  | Weighted median | 0.2557(8.2315e-02–0.7944) | 0.0183 |
|  |  |  | Inverse variance weighted | 0.4374(1.4526e-01–1.3174) | 0.1416 |
| Salted nuts intake | Female infertility | 23 | MR Egger | 1.0474(0.3183–3.4466) | 0.9399 |
|  |  |  | Weighted median | 1.0301(0.3761–2.8213) | 0.9539 |
|  |  |  | Inverse variance weighted | 1.0262(0.5001–2.1055) | 0.9436 |
| Salted peanuts intake | Female infertility | 14 | MR Egger | 2.4250(0.2406–24.4413) | 0.4681 |
|  |  |  | Weighted median | 0.6544(0.1689–2.5347) | 0.5394 |
|  |  |  | Inverse variance weighted | 0.7008(0.2046–2.4000) | 0.5714 |
| Spirits intake | Female infertility | 46 | MR Egger | 3.6797(1.2266–11.0383) | 0.0250 |
|  |  |  | Weighted median | 1.2835(0.6350–2.5944) | 0.4868 |
|  |  |  | Inverse variance weighted | 1.4505(0.9084–2.3162) | 0.1192 |
| Tea intake | Female infertility | 40 | MR Egger | 1.1107(0.5076–2.4303) | 0.7941 |
|  |  |  | Weighted median | 1.0130(0.5921–1.7332) | 0.9621 |
|  |  |  | Inverse variance weighted | 0.7703(0.5383–1.1024) | 0.1536 |
| Unsalted nuts intake | Female infertility | 15 | MR Egger | 3.4345(0.2667–44.2158) | 0.3611 |
|  |  |  | Weighted median | 0.5946(0.1724–2.0508) | 0.4105 |
|  |  |  | Inverse variance weighted | 0.7354(0.2638–2.0498) | 0.5568 |
| Unsalted peanuts intake | Female infertility | 45 | MR Egger | 2.9394(0.3722–23.2102) | 0.3121 |
|  |  |  | Weighted median | 2.5533(0.6115–10.6613) | 0.1985 |
|  |  |  | Inverse variance weighted | 1.6396(0.5878–4.5735) | 0.3447 |
| Yogurt intake | Female infertility | 10 | MR Egger | 1.2509(0.2427–6.4472) | 0.7966 |
|  |  |  | Weighted median | 1.1808(0.5794–2.4065) | 0.6471 |
|  |  |  | Inverse variance weighted | 0.8448(0.4953–1.4408) | 0.5359 |
| Never eat eggs, dairy, wheat, sugar: Dairy products | Female infertility | 5 | MR Egger | 0.0108(1.2393e-15–9.4666e+10) | 0.7853 |
|  |  |  | Weighted median | 0.1639(2.8953e-07–9.2822e+04) | 0.7890 |
|  |  |  | Inverse variance weighted | 0.0356(1.2139e-06–1.0447e+03) | 0.5251 |
| Never eat eggs, dairy, wheat, sugar: Eggs or foods containing eggs | Female infertility | 8 | MR Egger | 2.6965e-03(3.4980e-20–2.0787e+14) | 0.7756 |
|  |  |  | Weighted median | 3.9805e-01(3.3362e-06–4.7491e+04) | 0.8772 |
|  |  |  | Inverse variance weighted | 4.4708e+02(1.1338e-02–1.7628e+07) | 0.2583 |
| Never eat eggs, dairy, wheat, sugar: I eat all of the above | Female infertility | 28 | MR Egger | 0.2008(2.6866e-05–1501.3082) | 0.7274 |
|  |  |  | Weighted median | 1.4393(1.6983e-01–12.1985) | 0.7383 |
|  |  |  | Inverse variance weighted | 0.4490(8.7929e-02–2.2936) | 0.3359 |
| Never eat eggs, dairy, wheat, sugar: Sugar or foods/drinks containing sugar | Female infertility | 22 | MR Egger | 6.6379(1.2205e-05–3.6099e+06) | 0.7818 |
|  |  |  | Weighted median | 1.9112(1.8452e-01–1.9796e+01) | 0.5870 |
|  |  |  | Inverse variance weighted | 2.3622(4.3648e-01–1.2784e+01) | 0.3183 |
| Never eat eggs, dairy, wheat, sugar: Wheat products | Female infertility | 7 | MR Egger | 3.2053(0.0033–3029.8744) | 0.7524 |
|  |  |  | Weighted median | 3.2666(0.0216–491.8593) | 0.6435 |
|  |  |  | Inverse variance weighted | 9.4714(0.1046–856.8819) | 0.3279 |

**Supplementary Table 4.** Heterogeneity and pleiotropy between diets and female infertility assessed using different methods.

| Exposure | Outcome | Methods | Heterogeneity | | Pleiotropy | | | MR-PRESSO |
| --- | --- | --- | --- | --- | --- | --- | --- | --- |
|  |  |  | Q | P value | Egger_intercept | SE | P value | P value |
| Bacon intake | Female infertility | MR Egger | 10.7792 | 0.3749 | 0.0057 | 0.0254 | 0.8268 | 0.4620 |
|  |  | IVW | 10.8336 | 0.4573 |  |  |  |  |
| Beef intake | Female infertility | MR Egger | 15.2764 | 0.2266 | -0.0072 | 0.0398 | 0.8581 | 0.4170 |
|  |  | IVW | 15.3188 | 0.2878 |  |  |  |  |
| Beer intake | Female infertility | MR Egger | 17.3581 | 0.4303 | -0.0035 | 0.0219 | 0.8723 | 0.5940 |
|  |  | IVW | 17.3852 | 0.4967 |  |  |  |  |
| Bread intake | Female infertility | MR Egger | 38.0562 | 0.0456 | -0.0305 | 0.0235 | 0.2068 | 0.0810 |
|  |  | IVW | 40.6131 | 0.0339 |  |  |  |  |
| Cereal intake | Female infertility | MR Egger | 52.8611 | 0.0439 | 0.0019 | 0.0180 | 0.9139 | 0.0840 |
|  |  | IVW | 52.8780 | 0.0549 |  |  |  |  |
| Cheese intake | Female infertility | MR Egger | 46.8312 | 0.8740 | -0.0217 | 0.0122 | 0.0810 | 0.7980 |
|  |  | IVW | 49.9826 | 0.8183 |  |  |  |  |
| Coffee intake | Female infertility | MR Egger | 56.5617 | 0.0158 | -0.0028 | 0.0091 | 0.7554 | 0.0100 |
|  |  | IVW | 56.7164 | 0.0200 |  |  |  |  |
| Cooked vegetable intake | Female infertility | MR Egger | 10.0473 | 0.8167 | 0.0630 | 0.0546 | 0.2663 | 0.7920 |
|  |  | IVW | 11.3803 | 0.7854 |  |  |  |  |
| Dried fruit intake | Female infertility | MR Egger | 47.6843 | 0.1603 | -0.0195 | 0.0161 | 0.2353 | 0.1610 |
|  |  | IVW | 49.4608 | 0.1451 |  |  |  |  |
| Fresh fruit intake | Female infertility | MR Egger | 61.9233 | 0.1405 | 0.0037 | 0.0109 | 0.7338 | 0.1840 |
|  |  | IVW | 62.0653 | 0.1600 |  |  |  |  |
| Lamb intake | Female infertility | MR Egger | 28.0445 | 0.5155 | 0.0233 | 0.0172 | 0.1854 | 0.4850 |
|  |  | IVW | 29.8846 | 0.4715 |  |  |  |  |
| Milk intake | Female infertility | MR Egger | 12.9464 | 0.7947 | 0.0002 | 0.0141 | 0.9868 | 0.8330 |
|  |  | IVW | 12.9467 | 0.8412 |  |  |  |  |
| Non-oily fish intake | Female infertility | MR Egger | 8.0540 | 0.5287 | 0.0417 | 0.0310 | 0.2117 | 0.4800 |
|  |  | IVW | 9.8611 | 0.4527 |  |  |  |  |
| Oily fish intake | Female infertility | MR Egger | 49.9321 | 0.7937 | 0.0154 | 0.0118 | 0.1962 | 0.7250 |
|  |  | IVW | 51.6405 | 0.7703 |  |  |  |  |
| Pork intake | Female infertility | MR Egger | 4.1896 | 0.9640 | 0.0006 | 0.0365 | 0.9851 | 0.9800 |
|  |  | IVW | 4.1899 | 0.9797 |  |  |  |  |
| Poultry intake | Female infertility | MR Egger | 3.3873 | 0.6405 | -0.2428 | 0.2309 | 0.3412 | 0.6330 |
|  |  | IVW | 4.4924 | 0.6103 |  |  |  |  |
| Processed meat intake | Female infertility | MR Egger | 22.0502 | 0.3966 | -0.0353 | 0.0234 | 0.1464 | 0.3750 |
|  |  | IVW | 24.4377 | 0.3247 |  |  |  |  |
| Raw vegetable intake | Female infertility | MR Egger | 10.0018 | 0.9035 | -0.0043 | 0.0267 | 0.8713 | 0.9500 |
|  |  | IVW | 10.0288 | 0.9309 |  |  |  |  |
| Red wine intake | Female infertility | MR Egger | 37.6456 | 0.0016 | 0.0546 | 0.0369 | 0.1579 | 0.0010 |
|  |  | IVW | 42.8082 | 0.0005 |  |  |  |  |
| Salted nuts intake | Female infertility | MR Egger | 18.4427 | 0.6208 | -0.0005 | 0.0123 | 0.9667 | 0.7380 |
|  |  | IVW | 18.4445 | 0.6793 |  |  |  |  |
| Salted peanuts intake | Female infertility | MR Egger | 18.8168 | 0.0644 | -0.0265 | 0.0215 | 0.2428 | 0.0700 |
|  |  | IVW | 21.4223 | 0.0445 |  |  |  |  |
| Spirits intake | Female infertility | MR Egger | 34.0103 | 0.8051 | -0.0151 | 0.0082 | 0.0734 | 0.7110 |
|  |  | IVW | 37.3803 | 0.7128 |  |  |  |  |
| Tea intake | Female infertility | MR Egger | 36.4820 | 0.4931 | -0.0078 | 0.0076 | 0.3095 | 0.4950 |
|  |  | IVW | 37.5433 | 0.4904 |  |  |  |  |
| Unsalted nuts intake | Female infertility | MR Egger | 19.0383 | 0.1219 | -0.0421 | 0.0328 | 0.2211 | 0.0820 |
|  |  | IVW | 21.4569 | 0.0904 |  |  |  |  |
| Unsalted peanuts intake | Female infertility | MR Egger | 47.8430 | 0.2825 | -0.0098 | 0.0154 | 0.5260 | 0.2990 |
|  |  | IVW | 48.2977 | 0.3034 |  |  |  |  |
| Yogurt intake | Female infertility | MR Egger | 6.0369 | 0.5354 | -0.0139 | 0.0281 | 0.6349 | 0.8350 |
|  |  | IVW | 6.2831 | 0.6155 |  |  |  |  |
| Never eat eggs, dairy, wheat, sugar: Dairy products | Female infertility | MR Egger | 2.6257 | 0.4529 | 0.0027 | 0.0333 | 0.9387 | 0.6470 |
|  |  | IVW | 2.6326 | 0.6210 |  |  |  |  |
| Never eat eggs, dairy, wheat, sugar: Eggs or foods containing eggs | Female infertility | MR Egger | 9.8325 | 0.1318 | 0.0265 | 0.0419 | 0.5507 | 0.1660 |
|  |  | IVW | 10.4869 | 0.1626 |  |  |  |  |
| Never eat eggs, dairy, wheat, sugar: I eat all of the above | Female infertility | MR Egger | 28.0353 | 0.2144 | 0.0048 | 0.0266 | 0.8587 | 0.2320 |
|  |  | IVW | 28.0748 | 0.2568 |  |  |  |  |
| Never eat eggs, dairy, wheat, sugar: Sugar or foods/drinks containing sugar | Female infertility | MR Egger | 16.4403 | 0.6277 | -0.0054 | 0.0351 | 0.8787 | 0.6940 |
|  |  | IVW | 16.4642 | 0.6874 |  |  |  |  |
| Never eat eggs, dairy, wheat, sugar: Wheat products | Female infertility | MR Egger | 5.1744 | 0.3949 | 0.0063 | 0.0153 | 0.6940 | 0.5740 |
|  |  | IVW | 5.3543 | 0.4992 |  |  |  |  |

Supplementary Table 5. The results of Mendelian randomization analysis after detection and exclusion of outliers by the MR-PRESSO test.

| Exposure | Outcome | Removed SNPs | Methods | OR (95% CI) | P-value |
| --- | --- | --- | --- | --- | --- |
| Coffee intake | Female infertility | rs13163336 | MR Egger | 0.8650(0.3138–2.3845) | 0.7809 |
|  |  |  | Weighted median | 1.0122(0.5220–1.9626) | 0.9713 |
|  |  |  | Inverse variance weighted | 0.7922(0.4800–1.3074) | 0.3622 |
| Red wine intake | Female infertility | rs62573521 | MR Egger | 0.0647(0.0003–12.4992) | 0.3241 |
|  |  |  | Weighted median | 0.2578(0.0868–0.7656) | 0.0146 |
|  |  |  | Inverse variance weighted | 0.5178(0.1887–1.4203) | 0.2011 |

Supplementary Table 6. The results of Multivariate Mendelian randomization analysis.

| Exposure | Outcome | Methods | OR (95% CI) | P-value |
| --- | --- | --- | --- | --- |
| Adjustment of folate, Vitamin D, Vitamin B12, and cooked vegetable intake | | | | |
| Folate | Male infertility | IVW | 21.3096(0.9287–488.9185) | 0.0556 |
| Vitamin D | Male infertility | IVW | 0.1565(0.0004–54.2426) | 0.5342 |
| Vitamin B12 | Male infertility | IVW | 0.3893(0.0010–147.3725) | 0.7554 |
| Cooked vegetable intake | Male infertility | IVW | 23.3369(0.6331–860.0986) | 0.0869 |
| Adjustment of zinc, selenium, and cooked vegetable intake | | | | |
| Zinc | Male infertility | IVW | 2.6595e-04(1.0456e-19–6.764202e+11) | 0.6492 |
| Selenium | Male infertility | IVW | 2.426272e+15(4.2902e-23–1.372134e+53) | 0.4244 |
| Cooked vegetable intake | Male infertility | IVW | 34.3751(2.1094–560.1708) | 0.0130 |
| Adjustment of zinc, selenium, and processed meat intake | | | | |
| Zinc | Female infertility | IVW | 9.9225e+01(3.8379e-03–2.565339e+06) | 0.3751 |
| Selenium | Female infertility | IVW | 7.4474e-12(5.4780e-22–1.0125e-01) | 0.0314 |
| Processed meat intake | Female infertility | IVW | 3.7180e-01(1.8763e-01–7.3672e-01) | 0.0046 |
| Adjustment of monounsaturated fatty acids, polyunsaturated fatty acids, saturated fatty acids, and processed meat intake | | | | |
| Monounsaturated fatty acids | Female infertility | IVW | 1.0954(0.7144–1.6795) | 0.6759 |
| Polyunsaturated fatty acids | Female infertility | IVW | 0.9831(0.6208–1.5570) | 0.9424 |
| Saturated fatty acids | Female infertility | IVW | 1.0352(0.4427–2.4206) | 0.9362 |
| Processed meat intake | Female infertility | IVW | 0.4875(0.2600–0.9142) | 0.0251 |
